# Life on a knife edge: using simulation to engage young people in issues surrounding knife crime

**DOI:** 10.1186/s41077-018-0079-0

**Published:** 2018-10-04

**Authors:** H C Tribe, A Harris, R Kneebone

**Affiliations:** Centre for Engagement and Simulation Science, Imperial College London, London, UK

**Keywords:** Distributed simulation, Sequential simulation, Selective abstraction, Knife crime, Young people, Social inclusion

## Abstract

**Background:**

Knife-related behaviour among young people is an increasing social concern with a total of 35 teenagers killed by knife attacks in England in 2017. Distributed simulation has been shown to be a valid method of portable simulation for medical professionals; however, its role in delivering a socially educational message to members of the public has not been previously studied. This paper explores how the novel use of simulation could be used to address a serious social issue amongst young people at risk of criminal knife behaviour.

**Methods:**

A qualitative approach was used to study a two-part workshop attended by two groups of young people vulnerable to knife crime. Based on the concepts of sequential simulation and distributed simulation previously developed at the Imperial College Centre for Engagement and Simulation Science, the first part of the workshop showed the patient journey of a young man stabbed in the abdomen, attended by policemen and paramedics, followed by the participants witnessing a simulated emergency abdominal operation on a silicone model and concluded with a dialogue between the surgeon, the victim (who required an intestinal stoma as a result of the knife injury) and his mother. The second part of the workshop involved further discussion with the participants regarding the role of knives from the personal and community perspective. Visual data was recorded during the workshops and qualitative data obtained from group and individual interviews were thematically analysed.

**Results:**

A total of sixty teenagers aged 13–19 took part in the two workshops. The participant feedback suggested that the workshops provided a safe environment where young people could learn about and explore the consequences surrounding knife crime. Furthermore, participant recollection of key points was assessed between 4 and 6 weeks after the second workshop and the data suggested that the workshop could promote learning and a change in the participants’ knife-related behaviour in the future.

**Conclusions:**

The findings support further exploration of simulation as a modality for engaging young people about the issues surrounding criminal knife behaviour in a safe and cooperative environment. Moreover, the findings suggest that the workshop could be used as an educational tool that may facilitate behavioural change.

## Background

The use and carrying of knives by young people is a growing problem for our society. In August 2016, knife crime reached a 4-year high in London with 1749 recorded stabbings of people aged under 25, which is increased from 1719 in June 2012 [1]. Furthermore, children as young 6 years old are carrying knives for older youths [2]. Over the last several years, there have been many high-profile teenage murders and trusts have been set up in the name of the victims to address the problems associated with criminal knife behaviour. In 2010, Brooke Kinsella, the sister of the murdered teenager Ben Kinsella, was asked by the government to visit different schemes in England and Wales that addressed knife behaviour in young people. As a result of her recommendations [3], which included educating children about the dangers of knives in schools and at an earlier age, the government pledged £18 million to help fund local initiatives. As well as pledging financial resources, the government has set up task forces (Operation Blunt, Teal and Sceptre) to try and tackle the problem of taking knives off the streets. However, despite these programmes, knife crime is still an increasing problem.

One might suspect that the majority of criminal knife behaviour was gang related; however, Sir Bernard Hogan-Howe, the former Metropolitan Police Commissioner, has reported that 75% of knife-related injuries in those under 25 years old are not involved in gangs [4]. Furthermore, he stated that the reasons for this trend reversal are “self-protection, status, protecting criminal interests—such as a drugs business—and a culture of fear”. The role of status and fear in promoting the carrying of knives has been recognised for several years. The City Bridge Trust set up the community-based Fear and Fashion programme which aims to alter the attitudes young people have towards knives [5]. Young people are intimidated by the thought that other people carry knives and therefore feel the need to carry a knife for self-protection—the “fear” factor—and the act of carrying a knife can be socially admirable amongst peers—the “fashion” factor.

### Intervention

**Fig. 1:**
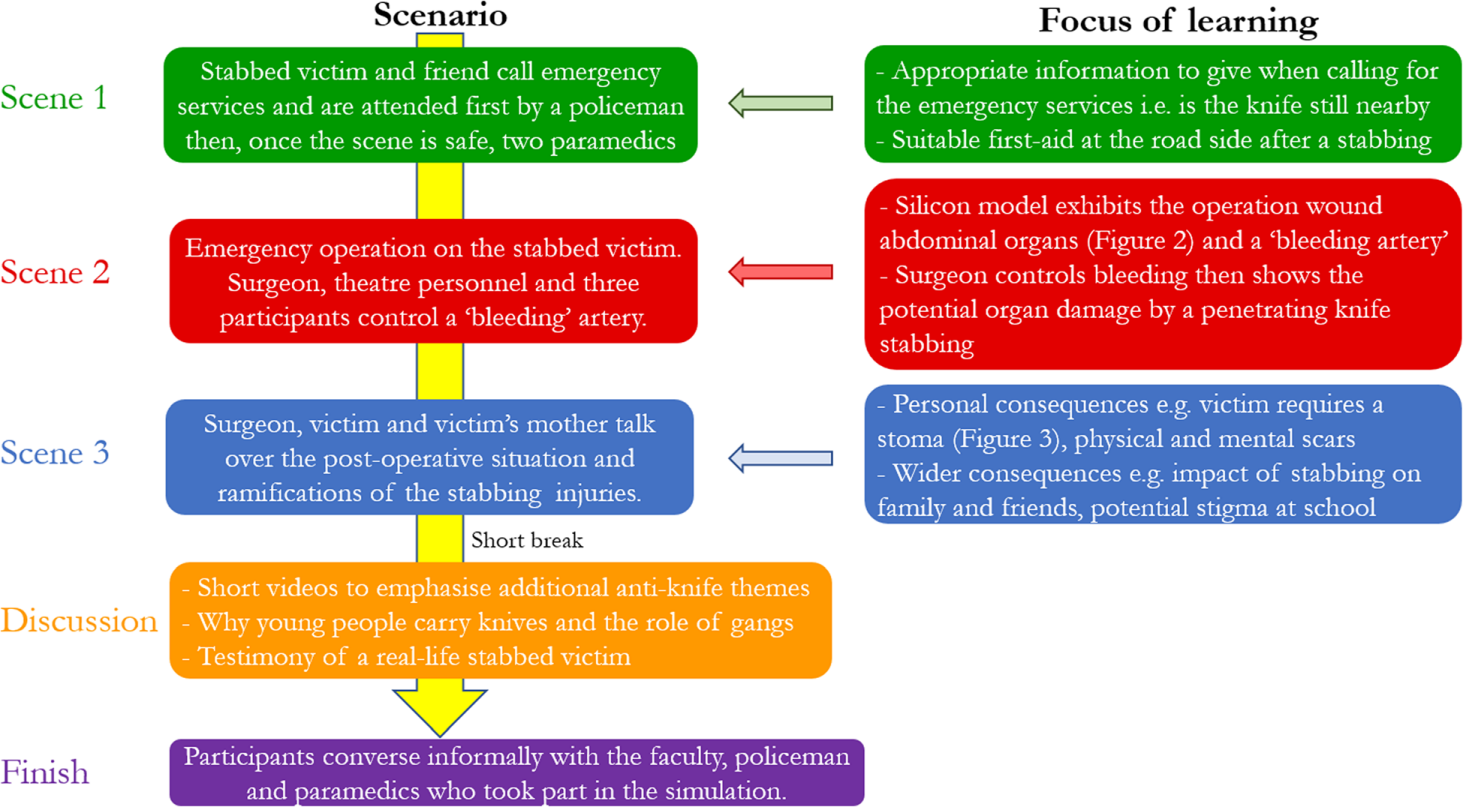
Workshop design. The workshop included a three-scene sequential simulation. After a short break, the workshop continued with a discussion of knife behaviour and the themes raised in the simulation

An additional reason for the attitudes of young people towards criminal knife behaviour is a lack of awareness of the legal and some of the medical consequences of a knife stabbing [6]. For example, teenagers may avoid stabbing in the heart to avoid a fatal wound but stab the buttocks instead, unaware that deep penetration may rupture major blood vessels resulting in death [7]. As a way of providing a better understanding about the ramifications of carrying and using knives, the team at the Imperial College Centre for Engagement and Simulation Science (ICCESS) created a mobile workshop consisting of a three-stage sequential simulation (SqS) followed by a group discussion (Fig. 1). Simulation, traditionally, involves a single, static clinical scenario. However, this does not accurately reflect a patient’s journey through a healthcare system. SqS recreates the longitudinal nature of healthcare by depicting specific scenarios of the patient’s healthcare journey [8–11]. This approach allows the participants who observe the SqS to gain a wider and more complete understanding of a patient’s clinical experience.

While SqS has not been principally designed for portability, the concept of distributed simulation (DS) has been developed by the ICCESS as a method of providing a portable, affordable and valid simulation set-up, which is away from formal indoor simulation centres [12, 13]. The over-riding premise of DS is not to recreate every small detail of reality, but, through a method of “selective abstraction”, key aspects of the real clinical environment are retained and sufficient visual, auditory and kinaesthetic cues are presented to the user to allow immersion into the simulation [14]. The reframing of simulation in healthcare allows for a participant to appreciate the clinical context of the simulation but through a portable and low-cost set-up [15].

Taking the concepts of SqS and DS further, the primary aim of this study was to examine the feasibility of using a mobile platform to deliver an educational workshop. The creation of a completely mobile set-up would enable the workshop to be entirely self-sufficient and run without any ancillary support, thus allowing the workshop to be taken to “hard-to-reach” groups who would not normally access simulation. A secondary aim was to ascertain whether information delivered at the time of the workshop could be retained and thirdly, whether the workshop could facilitate a behavioural change in the participants concerning knife-related activities.

Mobile simulation healthcare vehicles exist [16–19], but, to our knowledge, there are no published reports within the English language that describe a mobile surgical simulation designed to educate lay young people about an important social issue.

## Methods

The workshop was completed on two separate occasions. The first workshop was repeated four times on the same day and included 48 pupils (aged 13–14 years) from seven state-run schools in the area around Wimbledon, London. The participants were part of an “Aspirations Project” that was set up to help educationally and socially disadvantaged pupils reach their potential. The workshop was held in the grounds of King’s College School, Wimbledon, as this was the usual meeting point for the project. This first workshop was evaluated primarily from a feasibility perspective. The second workshop was with 12 teenagers (aged 14–19 years) from the “Saracens HITZ programme”, which is a social inclusion programme using the sport of rugby as a springboard to teach the attendees life skills. This group was chosen as it represented our preferred audience better as these participants conceivably had a greater exposure to criminal knife behaviour and could potentially benefit more directly from the workshop. This second workshop was held in the same park in East London where the group usually met. This ensured that the participants were in their own space and avoided any alienation that might occur if the workshop was held in a more formal location. The simulation and discussion elements of the workshop lasted approximately 45 min with a 30-min break between the simulation and discussion and then another 30 min at the end of the workshop for the participants to talk informally with the faculty members as desired.

**Fig. 2:**
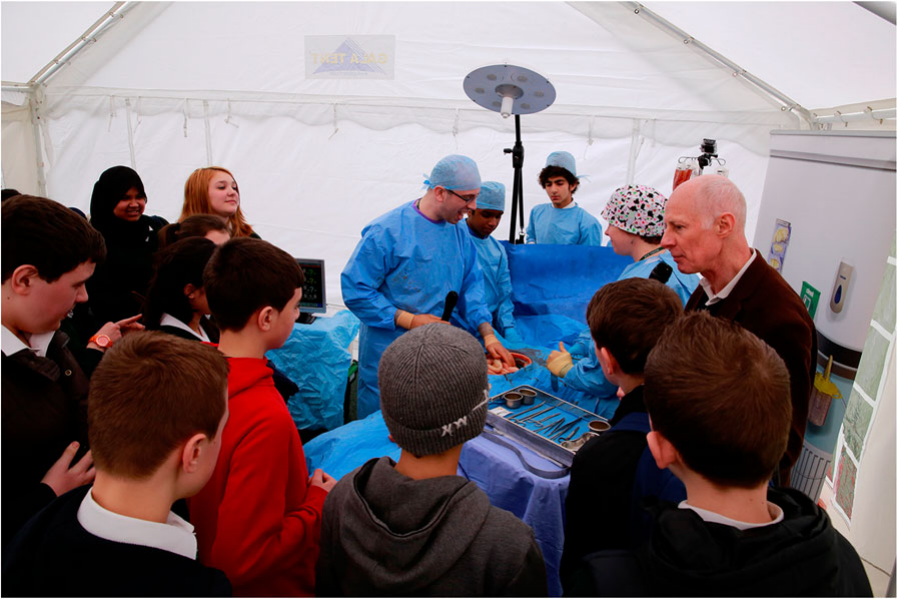
A photograph taken at the first workshop showing the abdominal operation in progress with the operating theatre team (including participants) dressed in blue, observed by the participants

**Fig. 3:**
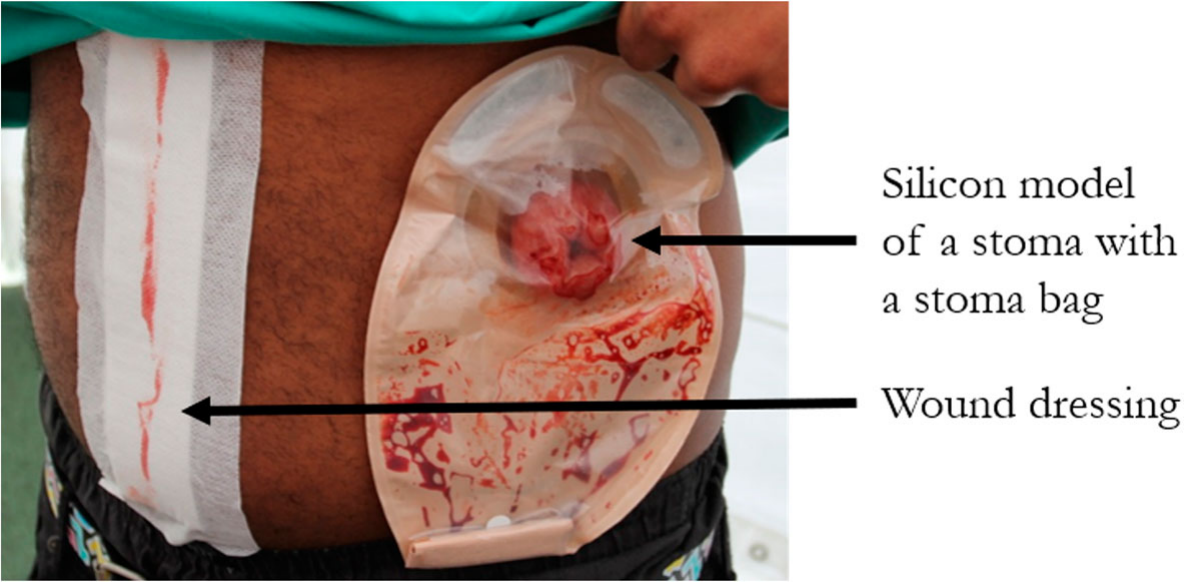
A photograph showing the stoma (a surgical procedure where the intestines are brought out on to the surface of the skin due to intestinal damage) and a stoma bag with a surgical dressing indicating the abdominal operation wound

**Fig. 4:**
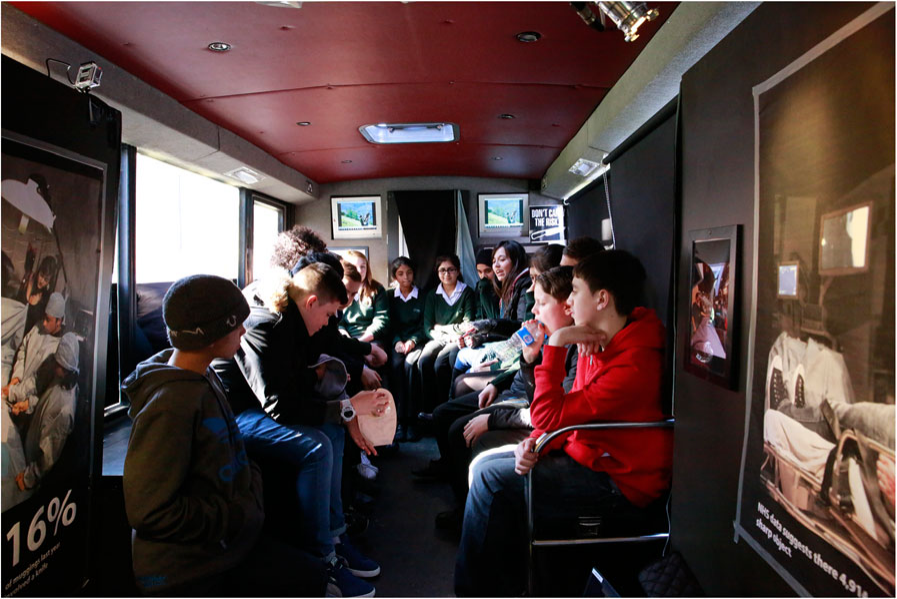
A photograph taken at the first workshop showing the inside of the converted single-decker bus. Facts and statistics related to criminal knife behaviour were presented on the walls, and the seating area was in a horse-shoe shape to aid discussion

For the first workshop, the three-part sequential simulation, with the silicon models, was held in a 6 m × 6 m marquee (Figs. 2 and 3). The discussion element to the workshop was held in a converted single-decker bus, which was parked immediately next to the marquee. The bus had a horseshoe-shaped seating area and large posters of knife crime-related facts and statistics placed on the walls (Fig. 4). The bus was chosen as it fulfilled a multipurpose role—a personnel and equipment transportation vehicle, a faculty preparation area and a distinct area from the marquee that allowed a physical separation between the simulation and the discussion elements of the workshop. This separation also permitted the simulation and the discussion to be run simultaneously. The same set-up was planned for the second workshop, but the bus incurred a last-minute technical fault; consequently, both the simulation and the discussion were held in the marquee.

For this exploratory study, a qualitative design was chosen. During the workshops, data was captured through photographic and video recordings, observations and field notes. After both workshops, round table and one-to-one feedback discussions were held with members of the faculty (a total of eight members). For the participants of the first workshop, data was also obtained via a semi-structured discussion with five participants from one of the schools. For the schools where no direct follow-up communication with the participants was possible, feedback was obtained indirectly from a teacher of the six other schools, who summarised the participants’ responses in an email communication.

In order to address the secondary and tertiary aims of knowledge retention and behavioural change, three educational and two behavioural objectives, respectively, were identified prior to the workshops (Table 1). The learning associated with each educational objective was delivered during the simulation, and knowledge retention was assessed during the one-to-one semi-structured interviews after the second workshop. The behavioural objectives were also addressed during these interviews.

**Table 1:** The education and behavioural objectives that underpinned the design of the workshop

Educational objective 1	Have the participants remembered that when they are calling for an ambulance they should say whether the scene of the knife violence is safe or not?
Educational objective 2	Have the participants remembered any personal physical or psychological consequences of a knife wound to the abdomen?
Educational objective 3	Have the participants remembered any of the wider consequences of knife-related behaviour?
Behavioural objective 1	Have the participants’ attitudes towards knife violence changed since attending the workshop?
Behavioural objective 2	Have the participants experienced or are likely to experience any behavioural change since attending the workshop?

All recorded discussions were transcribed, and themes were identified by triangulating the data from the recorded discussions, the observational data and the indirect data from the supervisors of the participants. The interview data from the participants of the second workshop was transcribed and analysed using Nvivo 10 (QSR International, Melbourne, Australia).

## Results

### Thematic analysis

For the first part of the workshop evaluation, data was collated from both workshops to create a thematic analysis of the feasibility, structure and content of the workshop as a whole. The data revealed four main themes: the set-up, the simulation, the discussion and the participants. Secondary subthemes were identified within the four main themes and are presented in Table 2.

**Table 2:** Overview of the identified themes and subthemes

Themes	Set-up	Simulation	Discussion	Participants
Subthemes	Feasibility Contingency	The operation Performance Scenario immersion	Location Content Real-life stabbed victim	Number Target audience Interaction Impact Unexpected behaviour

#### The set-up: feasibility

The opinion from the faculty was that the workshop was feasible in its current format. The converted bus performed multiple roles, although a lack of space was identified to be a problem at times. The marquee space was judged to be acceptable considering the financial cost and the time required for setting up; however, temperature control (related to the external ambient environmental temperature) was problematic. Obtaining permission from the local government authority to drive the bus on to a public park, although challenging, was possible.

#### The set-up: contingency

As part of the planning process, contingency options were prepared. As one faculty member pointed out, “you have got to be covered with all bases” (Faculty 7) when planning a mobile simulation as problems are likely to occur. For example, due to a technical fault with the converted bus 2 days prior to the second workshop, an alternative transportation vehicle was used, and the workshop was redesigned.

#### The simulation: the operation

The surgical silicon model proved particularly memorable, being highlighted at both time points. Additionally, the participants who volunteered to assist during the operation found the opportunity to don surgical gowns and gloves enjoyable and immersive.

“Probably still the favourite part of it is just, like, seeing the dummy and being involved in, like, just putting on the whole surgical stuff…” (2.2)

#### The simulation: performance

There was a general appreciation of the performance nature by both the actors and the police and healthcare personnel. The emotional aspects of the simulation were valued and the fact that simulation was delivered by not only professional actors.

“The use of real police/medics I think was a clever touch and made things more believable for the girls.” (Email 2)

However, there was a learning curve for some of the emergency service personnel and the difficulty of adjusting to a performance role was recognised.

“And there’s that issue of when you’re getting people who are drawing on in their own experience to play a role when he’s a policeman but he’s not being a policeman, he’s playing being a policeman.” (Faculty 2)

#### The simulation: scenario immersion

Despite the reduction in contextual immersive cues for this simulation compared to previous ICCESS simulation events, the faculty thought that a sufficiently immersive environment was retained. The key engagement hook was the silicon model of the wound as the participants “are all looking at the guts [of the silicon model], everything else is window dressing” (Faculty 4).

#### The discussion: location

For the first workshop, the discussion was held in a converted single-decker bus, whereas for the second workshop, it was held within the marquee due to technical reasons. Each location received mixed reports. The bus was physically separate from the marquee which was considered as either an advantage or disadvantage from an engagement point of view. Space and temperature within the two locations were also raised by the faculty and participants.

#### The discussion: content

Not all the content within the discussion section of the first workshop was well received with several participants admitting confusion to some of the themes examined. Consequently, the content was altered for the second workshop and was judged successful.

“The short film…anti-knife thing that we watched on the TV was really interesting because it made you think about how, if you go away for what you’re doing because your, like, friends pushed you into it, their life carries on.” (1.1)

#### The discussion: real-life stabbed victim

Members of the faculty and one participant from the first workshop remarked that the testimony from the real-life victim of a knife stabbing would be a powerful way of delivering an anti-knife message. For the second workshop, a man who had been stabbed in real life gave his personal story, which was well received and clearly impacted on many of the participants.

“Probably the interview…helped me to see what the consequences were and the impact on him and the fact he was so open about it…” (2.4)

#### The participants: number

The large number of participants was identified as a problem for the first workshop. In order to accommodate all the participants, they were split into four groups based on their school, with the workshop repeated for each group. The large number of participants in one of the groups was mentioned as a limitation due to a lack of seating space within the bus. Additionally, it was remarked that too many people at one time would reduce the impact of the simulation.

#### The participants: target audience

The faculty debated extensively who the most appropriate audience for this workshop is. The overall impression was that any participant of teenage years would benefit, but those most at risk of criminal knife behaviour would be the most appropriate. After the first workshop, the faculty thought that the participants did not benefit as much as anticipated with some “more engaged than others” (Faculty 3). This impression was mirrored in an email from one of the teachers, who stated that “the girls found it useful and wished they had got even more involved.” (Email 2).

For the second workshop, the faculty agreed that this group was more suited. There was recognition that the participants were attending of their own free will and as the workshop was located away from a formal location, they did not have to “toe-the-line quite so much.” (Faculty 4). Therefore, the workshop was much more on “their turf, their terms.” (Faculty 3). However, the participants’ personal experience with criminal knife behaviour was less than expected.

#### The participants: interaction

The participants from the first workshop were much more passive than those from the second. As the participants of the first workshop were not at their usual school, there was an impression that they felt “a bit of a cuckoo in the nest” (Faculty 2). Additionally, the participants had only met as a group on one previous occasion and “so maybe there was an element of shyness” (Faculty 3) which affected their responses.

#### The participants: impact

The emotional aspect of the workshop on the participants of both groups was evident. However, this facet of the workshop was felt more acutely by the participants of the first workshop.

“…some were affected by the visual and emotional impact of the experience, more than pupils from the other schools in our group (one of our pupils fainted and one left the drama) I felt some of our pupils had a big emotional journey.” (Email 3)

#### The participants: unexpected behaviour

A member of the faculty reported that the behaviour of the participants of the second group was more receptive than expected. He had worked with the participants for several years and found the behaviour expressed during the simulation illuminating.

“…to see him in that light was very interesting for me because it gave a breath of knowledge, in terms of the sort of person that he is and how I can deal with him in the future. He showed a sensitive side of him which I haven’t seen before.” (Faculty 8)

### Educational and behavioural objectives

In order to evaluate the longer term and wider impact of the workshop, one-to-one semi-structured interviews were held with a total of eight of the participants of the second workshop. In the original methodology, five participants would be interviewed 1 week after the second workshop (time point 1, TP1) and then the same participants would be interviewed 1 month later (time point 2, TP2). However, logistically, this proved to be impossible due to the continued non-attendance of three of the original interviewees. Therefore, three additional participants were chosen for the interviews at TP2. The logistical delay led to this round of interviews being conducted between 4 and 7 weeks after the workshop. Table 3 lists the codes for the interviewees at the two different time points and includes demographic information.

**Table 3:** The interviewee codes at the two time points accompanied by demographic information

Interviewee code at time point 1	Interviewee code at time point 2	Sex	Previous criminal knife behaviour experience
1.1	2.1	Male	Attended an educational workshop
1.2	2.2	Female	Nil
1.3		Male	Nil
1.4		Male	Attended an educational workshop
1.5		Male	Personal victim experience
	2.3	Male	Personal observer experience
	2.4	Male	Attended an educational workshop
	2.5	Male	Personal observer experience

Analysis of the data revealed good evidence that all three of the educational objectives and one of the behavioural objectives had been met (Table 4). Participants were able to remember the information they should report when calling the emergency services; however, some specifics were forgotten by TP2. Recall of the personal consequences of a knife wound was strong at TP1 and TP2. For the wider consequences, recall was strong at both time points; however, some of the responses at TP2 did not appear to relate specifically to the information given during the simulation.

**Table 4:** Analysis of data with respect to the five learning objectives of the workshop

Objectives		Summary	Quotes from transcript
Educational	1. Emergency services call information.	Three of the five participants explicitly described the correct response within their interviews at TP1. At TP2, only one participant recalled the correct action while the other four only remembered the information obliquely.	“…if you hear of if you see, like, a knife crime being committed call for an ambulance and tell them if the scene’s safe or not.” (1.5) “…call 999 and tell them if, like, the area is clear from danger so the ambulance and that can get here quicker.” (2.5)
	2. Personal consequences of a knife wound.	Over both time points, the participants expressed an increased awareness of how an individual may be affected by a knife injury—physical, mental and legal consequences.	“…the poo bag, that’s never going to leave my memory [laughs].” (2.1) “…it’s a big thing when you get stabbed…physically and mentally because it does change your life.” (2.5)
	3. Wider consequences of criminal knife behaviour.	All participants at both time points were able to identify relevant wider consequences for the people around them, especially family members. However, some of the responses appeared to derive from instinct rather than relating to specific learning from the workshop.	“[A stabbing] affects the victim’s friends, family, the people who have to help him afterwards, like the social workers, doctors.” (1.5) “…antibiotics, going back and forth to the hospital, that sort of thing.” (1.2)
Behavioural	1. Any attitude change?	This objective was loosely achieved. The participants were not specifically asked their attitude towards criminal knife behaviour prior to the workshop therefore a direct pre-/post-workshop comparison could not be made. Furthermore, as identified in Table 3, three of the participants had previously attended an educational workshop and may have already had a responsible attitude towards knives that added bias to the data. Despite this, it was possible to identify a change in attitude in two participants.	“…all of the people that I hang around with already know, already know about the danger about stabbing because we already spoke about it before.” (1.3) “I think a bit more, even more cautious around knives and people that carry them.” (1.1) “…if you was [sic] to use the knife on someone you’d know the impact on that person so it, kind of discourages you to want to use a knife.” (2.4)
	2. Any behavioural change?	Across the two time points, the workshop appears to have had a clear impact on the behaviour on seven of the participants. Several of the participants expressed greater reticence now about stepping in if they saw a knife crime in action. There was a focus on first-aid related knowledge and dealing with the situation calmly. Two participants described intended changes in their behaviour towards other people who carried knives. These intentions were only expressed at time point 1.	“Before, I would have involved myself and tried getting the knife off of [sic] whoever but especially seeing the guy who got stabbed, it makes you think that being the bigger person by moving away is being the bigger person.” (2.3) “I did not know about the compression of the wound and to make it clot, so that’s one thing I’d do if I saw someone with a stab wound.” (2.4) “…if someone had a knife then, that I knew, I’d tell them that they should not be carrying it and I’ll explain to them why.” (1.3)

While there was consistent evidence for educational learning, evidence for behavioural change was varied. Only two of the eight interviewees expressed a change in attitude towards criminal knife behaviour, but seven of the participants expressed a change in behaviour with an increased reticence towards personal involvement and an increased willingness to aid stabbed victims and discourage others to carry knives in the future.

## Discussion

This study has shown that it is feasible to stage an SqS workshop away from the formality of a purpose-built simulation unit or an indoor space and that the participants were able to remember the educative content of the simulation, at least in the short term. Additionally, there is evidence to suggest that the event may be able to deter the participants from future criminal knife behaviour or from associating themselves with individuals who carry or use knives.

One key advantage of a mobile set-up is that the workshop could travel to participants as opposed to participants having to travel to the workshop. This increases the access of the workshop and facilitates engagement with people who would not normally attend a simulation. An additional advantage of the simulation travelling to the participants is that the location is likely to be familiar with the participants. This familiarity could reduce the anxiety participants may experience when faced with an unfamiliar and potentially authoritarian environment. Moreover, by entering the participants’ environment, the dynamic between the participants and the faculty may improve and lead to improved feeling of ease by the participants that could potentially initiate a higher level of engagement quicker. This concept is summarised by the phrase “their turf, their terms” and acts a useful reminder that a two-way learning interaction between the participants and the faculty can exist. Furthermore, the concept of “reciprocal illumination” can be used to frame the learning dynamic between all those who attend the workshop [20]. The faculty do not simply convey information to the participants but engage in an active learning process themselves. Although reciprocal illumination is not unique to this workshop, the mobile set-up flattens the hierarchy between the faculty and the participants and improves the “informal” nature of the learning environment.

The mobile set-up of the workshops did lead to feasibility difficulties that centred on logistical issues. Contingency planning was an important factor in the delivery of the workshops, although contingency planning is not unique to this study. Another critical component to the success of the workshops was the professional background of the faculty members. From the data, it was clear that the presence of genuine emergency professionals contributed to the realism of the workshop. The inclusion of a real-life knife victim increased the authenticity and potency of the second workshop. His testimony made a considerable impact on the participants and made them consider the implications of criminal knife behaviour from a fresh perspective. Another key feature of the workshops that had a powerful impact on the participants was the operating theatre simulation and the prosthetic intestinal stoma. These visual and auditory experiences yielded an unexpected sensitive side in some of the participants which could provide a springboard for future discussions surrounding the issues raised by the participants’ reaction to the simulation.

For both workshops, there was considerable debate among the faculty regarding the most appropriate participants. The faculty favoured targeting teenagers who are very likely to or have already committed criminal knife behaviour. However, other successful anti-knife projects have been conducted with participants as young as 8 years old, and Brooke Kinsella’s recommendations include working with young children [3]. Analysis of future workshops using participants of different ages and backgrounds could provide evidence to suggest to whom this type of intervention is of most value.

A further consideration is the number of participants who should attend the workshop at one time. To enable intimacy and engagement, a restricted number of participants attended the workshops in this study. This was primarily due to the simulated operation being relatively visually inaccessible, due to the silicon model being below standing eye-level. A live recording of the operation linked to an overhead monitor and alterations to the position of the operating table could engage a larger audience and potentially lead to a greater impact on the wider community. One of the key determinants of a successful initiative is its ability to reach the widest possible audience [21]. Therefore, future events must balance the benefits for the individual with the wider community.

### Limitations

Although rich data was obtained from a wide variety of sources, much of the data from the participants of the first workshop was obtained indirectly, weakening its validity. Additionally, for the second workshop, it was not possible to interview the same cohort at both time points; therefore, a direct comparison of the data collected at the two time points was not possible.

Four of the participants from the second workshop and an unknown number of participants from the first workshop had attended a previous event relating to knife crime. This background knowledge may have influenced their reactions to the workshop, either positively (reinforcing the information communicated) or negatively (by reducing the “novelty” of the workshop). Furthermore, we had expected that the participants for the second workshop to be poorly integrated within education or the workplace; however, in general, the participants were well integrated and only a few participants had personal experience of criminal knife behaviour. These two limitations relate back to the issue of whom would make the most appropriate target audience, and a considered approach will be required when deciding on the objectives of any future workshops.

The production of the workshop did involve a core faculty of many different people (compere, surgeon, anaesthetist, scrub nurse, two technical managers, three actors, paramedic and a policeman). Recruiting so many people could be difficult to replicate; however, all the faculty roles do not necessarily require genuine health or emergency service personnel. Furthermore, the workshop has only been completed in the Greater London area. Rolling out the workshop to a national level, perhaps in collaboration with hospital trusts, would provide further data to help refine the target audience.

## Conclusion

This paper has confirmed the feasibility of staging an SqS from a mobile platform. It has introduced a novel way to engage with young people regarding the personal and wider consequences of a socially critical issue. Furthermore, interview data from the participants taken after the second workshop suggest that learning had taken place and that future criminal knife behaviour could be reduced due the workshop. However, young people will always be free to make up their own decisions irrespective of attending an anti-knife workshop. Therefore, perhaps the most positive outcome possible from this type of intervention is that young people are equipped with the knowledge to encourage them to “think twice” before carrying out any future criminal knife behaviour and know what to do if involved as a bystander.

## Abbreviations

DS: Distributed simulation
ICCESS: Imperial College Centre for Engagement and Simulation Science
SqS: Sequential simulation
TP: Time point
